# Extra-Limites

**Published:** 1828-01-01

**Authors:** 


### EXTRA-LIMITES.

*Observations on Cullen's System and Doctrines of Fever, shewing the Necessity of their being abandoned by Teachers of the Principles and Practice of Physic, forming the third Communication on Fever.** By John Mackintosh, M.D. Acting Surgeon to the Ordnance in North Britain; Physician to the General Dispensary, Brown Square; and Lecturer on the Practice of Physic, &c. in Edinburgh.

It is the proud boast of the present æra, that the arts and sciences never flourished in such an extraordinary degree; while the uncertainty of physic, and the unsettled nature of medical opinions, are too notorious to require being insisted on in this place. For this difference there must be sufficient reasons;—one of the most obvious, it appears to me, is, the errors of Cullen's System; another is to be found in the present plan of medical education—but I must confine my present observations to the first-mentioned cause.

Let me ask, in what state would mechanics and chemistry now have been, if the improvers of these sciences had, like Cullen, assumed false facts upon erroneous theories, as the very basis of their systems?

Surgery obtained the start of physic, and still maintains the pre-eminence, in consequence of its professors proceeding upon wiser principles than those which have generally guided physicians. The public has seen this, and the consequence is, that, in London, surgeons of eminence derive a far greater part of their revenue from the practice of physic, than from that of pure surgery. In Edinburgh, the medical practice is enjoyed, not by pure physicians, but by surgeon-apothecaries, who have obtained the confidence of the public. From this class, the consulting physicians, strange as it may appear, are at present chosen, which is a proof that they possess the confidence of the rest of the profession. Some of these have their medical degrees from universities which formerly granted them without any examination; and these individuals have no reason to blush at this statement.

In the first communication, on Intermittent Fever,† I drew several conclusions from the facts previously detailed, the last of these I shall take the liberty of quoting in this place. " If these cases possessed no practical merit whatever, they promise to be productive of great advantage to medical science, by destroy-

* This paper was announced in the 93d No. of the Edinburgh Medical and Surgical Journal, to appear in the next No. in the following terms:—" The object of my next communication will be, to shew the necessity of abandoning the doctrines of Cullen, in teaching the principles and practice of physic." It was accordingly forwarded to Dr. Cragie, early in November, for that purpose: that gentleman mentioned to me, that he had taken the liberty to mark several passages, which he thought had better be erased, to which I freely assented. But, subsequently, he waited upon me, to state that he had since had a consultation with "*the other gentlemen*," and they wished to decline printing it, as it was too controversial for this Journal. At the same time, I think it due to Dr. Cragie to state, that he made many polite apologies for now declining that which he had previously promised more than once to do. It would have been more becoming, if the Editors had commenced the reformation with themselves. But I think no one can fail to discover the true motives of the professorial Editor, or Editors, after perusing the paper itself.

† Published in the 91st No. of the Edinburgh Medical and Surgical Journal.

ing the very foundation of the erroneous system of Cullen. The doctrines upon which this system is founded, have to this day bewildered old and young in the profession, who think and act only under the nod of authority. Cullen's System has been a great bar to all improvement in medicine, and this is the principal cause of the backward state of pathology in this country, when compared with the strides made in that department by our professional brethren in France."

The object of this present communication, is to enter more fully into this subject, and to prove how hollow this system really is.

The whole rests upon the fate of the second chapter, which treats of "the Proximate Cause of Fever"—or, in other language, of the nature and seat of the family of diseases denominated fever. The whole fabric is built upon principles which are there fully and minutely detailed. It is the foundation of the edifice; and if I can prove it to be unsound, the rest of the tenement must be deserted.*

I shall now, then, proceed to the task I have proposed to myself. "As the hot stage of fever is so constantly preceded by a cold stage, we presume (says Cullen) that the latter is the cause of the former, and, therefore, that the cause of the cold stage is the cause of all that follows in the course of the paroxysm."—*Paragraph* 34. This is certainly correct *when there is a cold stage;* we often, however, see cases where there is no rigor, or even chilliness, the excitement being the first part of the diseased action which can be detected. The practice of bleeding in the cold stage, and its success in stopping the paroxysm, proves that to be true, which was only stated by Cullen hypothetically. By that practice, the rigors cease on the instant, and neither of the subsequent phenomena generally takes place, if the patient is properly managed.

In the 35th paragraph, he attempts to explain the cause of the cold stage, by which I understand he means the first link in the chain of diseased action, and he concludes, that "*diminished energy of the brain*" is the cause. This is a slight specimen of his unphilosophical method of proceeding. He substitutes one difficulty for another, and, having given it a name, he assumes it as a fact in medicine. But he must have a cause capable of producing this diminished energy of the brain. Any cause will not do; and, therefore, he fixes upon one or other of several causes, which he supposes always to produce *sedative effects* on the body—namely, contagion, miasmata, cold, and fear. The application of any of these produces debility: and he also attempts to explain the relapses in fever, by the *re-application* of the same debilitating powers. According to Cullen, the cold stage is *always* preceded by debility, and the former *always* precedes the state of heat; *all* the symptoms denote weakness, which weakness is a proof of diminished energy of the brain.—*Par.* 37, *and last part of the* 35*th*.

Finding the doctrines extremely weak, Cullen is next obliged to seek support from an antiquated and mysterious old lady, commonly called "vis medicatrix Naturæ."—*Par.* 38 *and* 39. He then asserts, as he feels still in difficulty in pleading the case, that, during the cold stage, there is a *spasm of the extreme vessels, every where on the extremities of the arteries*, and he attempts to prove this assumption, by the well-known fact of the suppression of the excretions. But it is important that he should here speak for himself. "It is to be particularly observed that, during the cold stage of fever, there seems to be *a spasm* induced every where on the extremities of the arteries, and more especially of those upon the surface of the body. This appears from the suppression of all excretions, and from the shrinking of the external parts; and although this may, perhaps, be imputed, in part, to the weaker action of the heart, in propelling the blood into the extreme vessels, yet, as these symptoms often continue after the action of the heart is restored, there is reason to believe, that a spasmodic constriction has taken place; that it subsists for some time, and supports the hot stage; for this stage ceases with the flowing of the sweat, and the return of other excretions, which are marks of the relaxation of vessels formerly constricted."—*Par.* 40. In the 41st par. he speaks more distinctly of his idea of fever in the following terms.—

* It must be remembered, that Cullen's doctrines of fever and inflammation are nearly the same.

"That a spasm of the extreme vessels, however induced, proves an irritation to the heart and arteries; and that this continues till the spasm is relaxed or overcome;" and this spasm, therefore, he considers "as a principal part in the proximate cause of fever." Although puzzled as to "the cause of the spasm, whether it be directly produced by the remote causes of fever, or if it be only a part of the operation of the vis medicatrix Naturæ," but he embraces the latter opinion, and, by doing so, he is at least consistent, by substituting one difficulty for another, perhaps still more involved in mystery.

Before I make any farther remarks, I must quote the whole of the 46th, and the first part of the 47th paragraph, to shew my reader Cullen's distinct opinions. "Upon the whole, our doctrine of fever is explicitly this:—The remote causes, are certain sedative powers applied to the nervous system, which, diminishing the energy of the brain, thereby produces a debility in the whole of the functions, and particularly in the action of the extreme vessels. Such, however, is, at the same time, the nature of the animal economy, that this debility proves an indirect stimulus to the sanguiferous system; whence, by the intervention of the cold stage, and spasm connected with it, the action of the heart and large arteries is increased, and continues so, till it has had the effect of restoring the energy of the brain, of extending this energy to the extreme vessels, of restoring their action, and thereby especially *overcoming the spasm* affecting them; upon the removal of which, the excretion of sweat, and other marks of the relaxation of excretions, take place. This doctrine will, as I suppose, serve to explain, not only the nature of fever in general, but also the various cases of it which occur."

I shall now proceed to comment upon these passages, and I promise my reader to be very plain, and, I hope, intelligible in my statements, and as short as possible.

The term, "diminished energy of the brain," is so often used by Cullen, that I cannot avoid making one or two remarks upon it, and more particularly, as it is a principal part of the foundation of his doctrines. It is one of those vague terms employed in medicine, to express a great deal more than any one actually knows, but which explains nothing. It is one of those expressions, which satisfies the youthful mind by blinding it, and it prevents inquiry. We know nothing of the natural energy of the brain, or in what it consists, or how it is propagated; therefore we are in total ignorance of the precise meaning of "diminished energy." This plain objection to the term must shew the utter absurdity of assuming a mere idea as a fact, and placing it as the key-stone of any set of doctrines, to explain the nature and seat of a disease.

As to debility, which Cullen founds so much upon, I have only to observe, that he and many others confound debility, or actual weakness, with oppression and obstructed action, produced by functional diseases of various organs, and also by irregular determinations of blood, causing venous congestion in vital parts. It is not like the debility which takes place consequent upon great losses of blood, starvation, a protracted disease, an alteration of structure of any part of the body. It is mere oppression, produced by the lost balance between the arterial and venous systems. The moment that is restored, the overpowering sensations of weakness vanish, and by the agency, be it remembered, of a remedy *directly* debilitating.

It is, also worthy, of remark, that the sedative causes which Cullen has brought into play, produce nearly, if not entirely, the same consequences on the human body, as any external or internal irritation, the failure of any viscus in the performance of its proper functions. A scald, a blister, the long continuance of drastic purges, the sudden drying up of an old sore, or the recession of an old eruption, all these causes, and many others, occasionally produce febrile action: in fact, these opposite circumstances produce the same phenomena, exactly the same combination of symptoms, the diseases run the same course, and they have the same termination. It must, also, be mentioned, that the same phenomena are also produced by the direct application of a stimulant, with the exception of the cold stage. But it can easily be proved, that diametrically opposite causes will produce the same effects upon the human body. A piece of frozen mercury will occasion the same effects as red hot iron—and the sensation caused by both is exactly similar. The former acts by the sudden reduction of temperature of the part—the latter, by the sudden application of too much caloric. The limits of an article in a journal will not permit me to enlarge on each of these points; it is too evident they cannot be explained in a satisfactory manner by the doctrines of Cullen, however modified. I shall content myself with one or two examples. Let us observe what takes place where an important organ is impeded in its functions. If the organ is very important to life, all other organs, and, indeed, every part of the body, must be speedily affected, and a general commotion will take place in the system, unless the change has been effected very, very slowly indeed. If an organ is labouring under acute disease, every other organ must also speedily suffer, from the want of the necessary and natural supply of arterial blood, to enable it to perform its proper functions, and, hence, a general commotion in the system will follow: if it should be an excreting organ, something is retained in the blood, which acts like a poison on the rest of the system, and a general commotion will be excited, and kept up. Under all these circumstances, the brain may suffer, either in its functions or structure; but it does so along with the respiratory, circulating, biliary, and urinary organs; it is difficult, if not perfectly impossible, in most cases, to determine which part has been first affected: they are parts of the same machinery, they act and re-act on each other. Perhaps, generally speaking, the first circumstance observed in fever, is want of appetite, next to it nausea, and bad state of bowels, or a morbid condition of the urine. It is difficult to say which is the first link in the chain of diseased action, because the sudden disorder of one function, leads immediately to that of others.

Let me now ask, if it is necessary to go further into the explanation, to enable us to apply our remedial agents? I think not. But as many individuals, for whom I entertain a high degree of respect, think otherwise, and allege that they not only approve, but act upon Cullen's doctrines, I shall state a few cases, for the purpose of shewing the superiority of the common-sense views over the doctrines of Cullen, for all useful, and even scientific purposes.

A. B. having fallen into deep water, and being nearly drowned from long immersion, is at length rescued from a watery grave. A medical man is called to him, who finds the respiration almost suspended—the surface cold—no pulse at the wrist—total insensibility. The proper means are applied; the pulse rises, respiration becomes stronger, the heat of surface is restored, and, in fact, recovery goes on; but, in a few hours, a high fever supervenes. A practical man, who disregards all system, will say, "my patient is nearly dead; had he remained longer in the water, he must have died from want of breath. The blood has fled from the surface: there has been no loss of blood; the man has the same quantity of vital fluid in the body now, as before he fell into the water. Where has the blood gone to? Internal parts are gorged, the balance between the arterial and venous systems is lost: I must rouse the action of the heart by stimulants—restore the heat of surface by every possible means, and, having gained so much, the best thing I can do for my patient is to open a vein, to assist Nature in the struggle, by removing the congestion more quickly and effectually than she can do, if left to her own efforts."

The same phenomena are produced by breathing foul air. Two men, employed, at some distance below the surface, in cleaning out an old neglected well, came in contact with impure air; one fell a victim immediately, and was taken out quite dead—the other was extricated with difficulty; he was in a state of insensibility—the respiration was feeble and oppressed—the pulse so weak as not to be felt at the wrist—the surface cold; he had, in fact, the same symptoms as the individual who had been immersed in water. Proper remedies were applied, and he recovered, but experienced, also, a severe fever, which might have been called *a typhus gravior*.

In both these instances, the sudden impediment to the function of respiration produced the whole mischief. The causes were different, but the effects were similar in every respect. The common-sense views in these cases answer every practical purpose, but let us try to apply the doctrines of Cullen, and their folly will be too evident to require comment. "Certain sedative powers were applied in these cases: they produced diminished energy of the brain, causing debility, which (as a necessary consequence) produced spasm (*i.e.* undue contraction) of the extreme vessels every where; this *debility* produces *strength,* which rouses the action of the heart, this action of the heart continues till it has had the effect of restoring the energy of the brain—this energy of the brain will then be extended to the extreme vessels, restore their natural actions, overcome the *spasm* which had affected them, upon the removal of which the excretion of sweat will take place, and other marks of the relaxation of the excretories." Let us ask ourselves again, whether Cullen's doctrines, or the common-sense views, will be of most assistance in the treatment? I may here further remark, that those who still pursue Cullen's erroneous system will be in danger of puzzling themselves by endeavouring to find out the *causes,* which are named proximate, remote, and exciting. The proximate cause has already been discussed; it is, as I understand it, the disease itself. But they will try to discover the others. What made the man fall into the water? Was it from diminished energy of the brain, producing debility of the lower extremities in particular, or vertigo —or did he stumble by kicking his foot against a stone?—or, did a miscreant purposely push him into the water with criminal intent. In the other case they will try to enquire into the composition of the foul air. How did he go into the well? How long was he in it? Did he descend in a bucket, or by means of a ladder? These are all points of laudable curiosity no doubt; but, let me suppose that the medical man did obtain the precise information, what advantage would it be to the patient? We are called upon to treat the effects, and not the causes.

I have often seen fevers of the most severe kind, and I have several under my care at this present moment, produced by individuals bathing in the open sea, at a time, too, when in the enjoyment of perfect health. They remained too long in the water, which produced, in the first instance, a complete cold stage, by driving the blood from the surface, followed by reaction. Would it not be the height of folly to explain the nature and seat of the disease, by stating that there was, in the first instance, debility and diminished energy of the brain by the application of a sedative cause, producing spasm of the extreme vessels, &c. &c.

It has already been shown, that Cullen and others have always confounded real debility with oppression from obstructed action. But, granting that real debility is as regular an agent as Cullen could wish, in the production of fever, let his disciples answer three simple questions.

1*st.* Why does not debility, produced by so many different causes, invariably, or even pretty generally, induce the phenomena of fever?

2*dly.* At what period of fever does debility exist in the greatest degree? and, 3*dly,* If debility is admitted as a necessary part of the proximate cause of fever, or, in intelligible language, as a part of the disease itself, why should the combination of symptoms denominated fever ever end?

Cullen distinctly ascribes spasm of the extreme vessels to debility, and this spasm of the extreme vessels is also, according to him, a part of the disease itself. It is really curious to observe all that he and his disciples have attributed to debility and to spasm. His highly gifted and accomplished successor, Dr. Gregory, stated constantly in his lectures, that he had "no doubt the causes producing fever act first by producing debility, and accordingly we find, said he, that stimulants employed at this period have often produced good effects in checking the disease, while evacuations, such as blood-letting, which at another period of the disease might have been proper, if employed in this first stage, *never fails* to be attended with most dangerous consequences, or it is, to use the words of Celsus, '*hominem jugulare!!*'" "*The heart partakes of this debility also, and syncope frequently follows; this debility of the heart produces an accumulation of blood in the great vessels, and this occasions that unusual motion of the organs of respiration —yawning.*" "*The tremors depend partly, but not wholly, on debility.*" "*Difficulty of breathing is to be attributed to debility of all the muscles of respiration.*" "*Want of appetite, nausea, and vomiting, are owing to debility of the muscular fibres of the stomach.*" "*Costiveness is produced partly by debility and partly by spasm.*" "*Failure of attention and memory, and also delirium, are owing to debility.*" In fact, Cullen's definition of fever, founded not on matter of fact, but on theory, compelled him and his followers to adopt such views, and others quite as erroneous. "Progressis languore, lassitudine, et aliis debilitatis signis, pyrexia, sine morbo locali primario." Such is Cullen's definition of fever. Is it possible, after all the light which has been shed by morbid anatomy on the nature and seat of diseases, and the improvements which have taken place within these few years in pathology; I ask, is it possible, that candidates for the highest honour in medicine are compelled to burden their minds with the recollection of such trash, and to learn by heart Cullen's Nosology and Cullen's definitions? The symptoms of all diseases, and particularly of fever, have a very wide range of character; therefore definitions drawn from the symptoms cannot hold good. This method has been the means of fostering a symptomatical pathology; so much so, that I often see medical men, admirers of this system, pronounce a diagnosis upon the presence or absence of *one symptom;* and I see others fix upon one symptom, which they have treated as the disease itself. Two examples will suffice. Cough is not a disease, it is only a symptom of a number of diseases; yet, how often do we see cough treated in the exanthematous disease for instance, also in pertussis, in pure bronchitis, and in phthisis, in which affections the cough is a blessing to the patient, because speedy death would sometimes take place if the matter secreted in the air-passages were not quickly expelled. Neither is asthma a disease—it is only a symptom produced by several morbid conditions of the lungs, heart, and large vessels, &c. but the most frequent cause of asthma is chronic bronchitis. But, to proceed more closely with the doctrines of Cullen, I have to state that Dr Gregory used to say in his lectures, "The first stage of fever cannot be owing merely to debility, there must be something else. This I have no scruple in referring to *spastic stricture* of the extreme vessels." And again, after describing the symptoms, he stated, "I have no scruple in referring these symptoms to spasm of the extreme vessels; for, although many of these vessels are not above $\frac{1}{3000}$ part of an inch in diameter, and are not capable of admitting red particles, we must *suppose them supplied with muscular fibres, although this cannot be proved by anatomy, on account of their smallness.*" "There seems also (said he) to be a spasm of the small vessels of the internal parts, but, on account of the *congestion,* this is sooner relaxed than that of the external parts; and, from the relaxation, I think we may account for the vomiting of bile, &c. &c. *and even of black blood.*" He also attributed the thirst to spasm of the vessels of the mouth. "The urine, in the cold stage, is colourless, evidently owing to the constriction of the vessels, allowing none but the watery parts to pass."*

These passages were quoted with the view of shewing the low state of pathology in this University, even so lately as 1820, also the influence of authority in medicine, and that Dr. Gregory entertained the doctrines of Cullen to the fullest extent. Every one in practice must have seen cases of fever, in which copious perspiration continued from the beginning to the termination of the disease in death. Let me ask, what became of the doctrine of spasm of the extreme vessels in these cases? In intermittent fever the most certain method of producing a protracted disease is to allow the patient to sweat away in bed. Rheumatic fever, treated in the old way of sweating the patient for 14 days, did not, in most cases, reduce the febrile action; on the contrary, I have seen many individuals become more and more feverish under such management. Cullen himself, in treating of the practice of sweating in fever, and the arguments used against such a plan, makes the following observations:—"Thus, sweating employed to prevent intermittent fevers has often changed them into a continued fever, which is always dangerous. The utility of this practice (of sweating in fevers) is further doubtful, because sweating, *when it happens, does not always give a final determination;* as must be manifest in the case of intermittents, as well as in many continued fevers, which are sometimes in the beginning *attended with sweatings, that do not prove final; and, on the contrary, whether spontaneous or excited by art, seem often to aggravate the disease.*"—Par. 164.

---

* These statements are extracted from a beautiful copy of Dr. Gregory's Lectures in MS. taken by the late Dr. *Kenny.*

He then proceeds, in the following paragraphs, to state the circumstances in which sweating is to be avoided, and his reasons why it is injurious in some cases; and in the 166th paragraph he is compelled to make the following clumsy explanation:—"In these cases, it is probable, that either an inflammatory diathesis is produced, which increases the spasm on the extreme vessels, or that, from other causes, *the spasm is too much fixed* to yield easily to the increased action of the heart and arteries; and, upon either supposition, it must be obvious, that urging the sweat, is ready to produce a hurtful *determination to some of the internal parts*, and may be attended with very great danger." It is an axiom in life, that he who tells one falsehood is frequently under the necessity of sinning an hundred times to support the first error. In like manner, it will be seen, on a careful perusal of Cullen's whole system, that he is obliged, in every page, to commit outrages upon common sense to support his first error. Having formed certain opinions, he endeavours in vain to make the facts fit them. This is well seen, if his definitions of diseases are contrasted with nature at the bedside. I must crave a little patience from my readers, while I examine the plain unsophisticated meaning of the expression "*spasm of the extreme vessels*," as used by Cullen and others—the importance of this point will be presently admitted. I shall allow for the sake of argument, that the extreme vessels have mouths, even that they are $\frac{1}{3000}$ part of an inch in diameter, and that they are provided with muscular fibres, although even Dr. Gregory confesses no anatomist had ever seen them. Allowing all this, the term "*spasm of the extreme vessels*" surely means nothing else than *a morbid contraction* of these vessels. But Cullen, in the last sentence of the 44th paragraph, and likewise in the 45th also, speaks of an "atony of the extreme vessels." What is the meaning of this term? Is it not a *defect of muscular contraction* of the same vessels? Can these two opposite morbid conditions exist at the same time? Can a morbid contraction (spasm) and a morbid relaxation (atony) co-exist in the same vessels?—Gentle reader, if you feel your understanding rather insulted by these queries, the injury is not inflicted by me, but by Cullen and his disciples. Attend to Cullen's statement, first, with respect to the existence of spasm as an essential part of the proximate cause of fever. "The idea of fever, then, may be, that *a spasm of the extreme vessels*, however induced, proves an irritation to the heart and arteries, and that this continues till the *spasm is relaxed* or overcome," &c.—par. 41. And, again, in the 40th paragraph, as I have formerly shown, he speaks of an universal spasm on the extremities of the arteries during the cold stage; that this spasm continues and supports the hot stage, "for (says he) this stage ceases with the flowing of the sweat, and the return of other excretions, which are marks of the *relaxation of the vessels formerly constricted.*" But the following strange contradiction will be found in the last sentence of the 44th paragraph:—"From the whole we have now said on the subject, I think it is sufficiently probable, that the symptoms of anorexia, nausea, and vomiting, depend upon, and are a proof of, an *atony* subsisting in the extreme vessels on the surface of the body, and that this *atony*, therefore, now ascertained as a matter of fact, may be considered as a principal circumstance in the proximate cause of fever." This atony we suppose to depend upon a diminution of the energy of the brain, and that this takes place in fevers we conclude, not only from the debility prevailing in so many functions of the body mentioned above, but particularly from symptoms which are peculiar to the brain itself."

I deem it unnecessary to make any comment upon these strange and contradictory statements; they always surprised and confounded me when a student, and, however ingenious some people may still think the doctrines of Cullen, I cannot help expressing my honest conviction that they are downright nonsense. If Cullen meant any thing, it must be acknowledged to be erroneous, to say the very least of it. But, it may be said, he meant nothing. In either case, why should the profession cling to such a rotten system of pathology? It is necessary now to put two questions; 1st. to Teachers of Medicine. Why do you so generally laud this system, as being the most splendid edifice in medicine, and insist on students learning by heart that which you, if you are successful practitioners, must have forgotten or neglected?

*2dly*. To Professors of Universities—Why do you still require such imperfect proof of a candidate's fitness to receive a license to go forth among His Majesty's lieges, unless you distinctly avow, at once, that it is for the purpose of doing that which Charles the Second alleged of Willis?

It is truly surprising, that Cullen, well as he must have been acquainted with the history and progress of medicine, in all its branches, should have avoided the path which had formerly led Hippocrates, and more lately Sydenham, to so much reputation and eminence, that their works will never cease to be read with interest and advantage. But, above all, it is wonderful that he should not have profited by the errors of those who preceded him. We find that when Hippocrates fairly applied his mind more to the observation of the effects of outward circumstances and inward irritations on the constitution and functions of the human frame, than to the abstract study of hidden causes, his labours were crowned with success; and he was still more fortunate in his researches, when he avoided, not only the superstitions of the times, but, also, the properties and agencies of matter, with which the study of diseases had been previously blended. His want of success is equally well displayed, when, by taking another track, he allowed his imagination its full swing, and indulged in hypothetical opinions. We shall find the same contrast throughout the whole history of medicine, not only in different characters, but in the same individual. Let it never be said, however, that Cullen was not a great man; he had certainly no original turn of mind, all his doctrines are copied from Boerhaave, Stall, and Hoffman—but the beauties of his style of writing and his ingenuity in being able to prop up a false system by mysterious expressions and by special pleading, will entitle his work to a place in the libraries of our successors to the latest posterity, as a literary production. Every medical man should certainly be well acquainted with Cullen's writings, but the work I am now speaking of, instead of being the first book put into the hands of students, ought to be the very last, for the same reason that a child is always taught good principles in the first instance, before he can be supposed to discriminate between good and evil.

It may be said by practical men, that they themselves have long ceased to think of his doctrines, that they can scarcely recollect one of his definitions, and that I have been contending against a straw. It is not so. Residing, as I do, at one of the greatest Medical Schools in Europe, I have reason to know, that Cullen's opinions are taught and supported by almost every Teacher of Physic but myself—his symptomatical pathology is as much lauded as formerly. The best proof of the truth of this is to be found in the fact, that Cullen's definitions, symptomatology, and nosological arrangement, are required as tests of the Graduate's attention and proficiency; and if any one dared to state, that they were erroneous and, therefore, he did not think it necessary to burden his memory with them, it is questionable, however good his reasons might be, whether he would be licensed to kill and to cure by any University in the British Empire. It will, also, be found, that the learned and laborious work of Dr. Mason Good inculcates Cullen's doctrines, and, notwithstanding the studied alteration of terms, no one can fail to discover, in every page, an old friend with a new face. And, above all, at this very moment, two new editions of Cullen are actually advertised as "in the press, to be speedily published." I may mention that my attention was called, very forcibly, to the subject of this paper a few years ago, when I began to teach the Practice of Physic. It was then my intention to publish a new edition, as a text book for my pupils, but I soon abandoned the task, because the additions necessary to bring the work to the present state of science, would far have exceeded the bulk of the text. Another, and a far stronger objection to such an undertaking, appeared to me to be, the absurdity, in the present improved state of pathology, of re-printing such a mass of error, and, also, of following his classification of diseases.

It will doubtless be said that, although the doctrines of Cullen may be founded in error, yet his system, as a whole, is good—his description of diseases excellent, and his practice ought still to be pursued. My opinion is quite different on all these points, and I should be happy to join issue upon them at this opportunity, but the length to which this paper is already extended, forbids further comment than to beg the experienced pathological reader to refer to the work itself, with a view of ascertaining whether he can obtain sound views of the pathology of any disease from it. For example, let him peruse the Chapters on Phthisis—Pneumonic Inflammation—on Inflammatory Affections of the Abdomen—or, on Inflammation of the Brain:—To this last subject he devotes two pages and a half, while *Odontalgia* occupies no less than five!!

It may be asked by those who are not very well acquainted with the present state of advancement at which practical medicine has actually arrived, What good can accrue from the destruction of Cullen's System—have you a better to offer as an equivalent?

All Systems which have hitherto been promulgated are too arbitrary to be useful at the bed-side, which is the only true test. I confess that I have no system of my own to offer in place of that which I hope to destroy. It is, indeed, my earnest wish to see a pathological System of Medicine established, but it can never be the work of one man; fortunately, however, there is scarcely a corner in Europe or in America, in which there is not to be found some ardent cultivator of pathology, and it is by the efforts of the whole that the great and important task is to be completed. Rapid improvements have certainly been made of late years, but we must be patient in our investigation, for, until our knowledge of the structure and physiology of the nervous system is considerably advanced, we cannot hope to triumph over many difficulties which daily impede our progress. Under such circumstances, let us rather confess our ignorance and wait for an accumulation of facts, instead of using fallacious arguments, however ingenious, to bolster-up a false System.

31, *Albany Street, Edinburgh,*

*10th October,* 1827.

---

### POSTSCRIPT TO NO. XV.
*(December 20th, 1827.)*

### *Mrs. Denmark's Case—tying the Subclavian Artery for Aneurism of the Innominata.*—Mr. Wardrop's Reports to the Lancet.

The first of these reports* contains Mr. Wardrop's opinions, as to the principles upon which the operation is likely to effect a cure, and as to the originality of his own views on this subject.

2dly. The result of his examination of Mrs. D.'s chest, previously to performing the operation.

3dly. The steps of the operation itself, and its immediate effects upon the aneurismal tumour.

*Second Report.*† This is dated 9th August, and informs us, that the wound was then perfectly healed, and that the patient had gone to the country, having been "brought, by the operation, from a state of great suffering to one of comparative health."

*Third Report.* This consists of Dr. Barry's letter‡ to Mr. W. containing the notes which that gentleman had taken of the state of Mrs. D.'s heart and great arteries, on the 6th July, the day before the operation.

The next account we have of Mrs. D. is given in a speech,§ made by Dr. Barry, at the Westminster Medical Society, on the 10th November. After stating that the clavicular tumour had subsided, and that the pains of the head, neck, and

* Lancet, No. 202.
† Lancet, No. 206.
‡ Lancet, No. 208.
§ Lancet, No. 220.

shoulder had disappeared, he concludes by observing, that the dyspnœa still continues, and seems to express some doubts as to whether the span of life is likely to be prolonged, in this case, by the operation.

*Fourth Report.** "In this report, (the longest of the whole) Mr. Wardrop seems to consider Dr. Barry's modest statement as quite unworthy of the brilliancy of his success. He accordingly charges Dr. B. with having given a very imperfect account of the case, such as might mislead the public, and immediately publishes this fourth report, for the express purpose, as he declares, of saving the public from being misled. In this document, the history of the case is brought up to the 5th December. It is there stated, in the most unqualified terms, that, with the exception of a slight attack of bronchitis, which she had about a fortnight ago, the woman has continued to improve progressively ever since the operation, and that she is now perfectly cured.

We shall now briefly remark upon the different items of these reports.

First, then, the whole jet of the very prolix discussions which Mr. Wardrop has entered into in his first and fourth reports, as to the principles upon which he was led to perform the operation, is contained in the following sentence of Dr. Barry's speech.

"If, under these circumstances, (viz. aneurism of the innominata, with stoppage of the carotid) the subclavian be tied, a still less quantity of blood will be sent into the brachio-cephalic trunk, the motion is, consequently, diminished, and the cure, which Nature had commenced, is expedited."†

Mr. W. labours under a sad mistake, when, in order to shew the originality of his own views, he asserts that surgeons, up to the present day, "will insist," that a total stoppage of the circulation within the aneurismal bag, is indispensable to the formation of coagula, and consequent organization of fibrine. Cooper's Dictionary will settle this point.

Mr. W. takes to himself the credit of having noticed the imperfect dislocation and motion of the sternal end of the clavicle. The merit of this remark, if there be any in it, belongs to Dr. Barry alone.

With regard to the examinations of the chest, Mr. W. *after the most minute and careful examination with the stethoscope, and by percussion,* states, that the contents of the thorax were sound, except the innominata; yet he publishes Dr. Barry's notes, in which it is asserted that there was true aneurism of the aorta, as well as of the innominata. But, at the time these notes were sent to the Lancet, Dr. Barry's opinions, whether well founded or otherwise, could only add to the eclât of the operation, because the woman had been already reported as nearly, if not entirely cured.

We now come to the most important points of all, the final result of this operation, and the actual state of the patient who was the subject of it.

Immediately after we had seen Mr. W's. last report, we sought for, and found out, Mrs. Denmark. We saw her on the 12th December, and afterwards on the 16th. She was still alive, and may continue to live for some days. We examined her chest, and observed the symptoms under which she laboured with much attention. *There is aneurism of the aorta, in a very advanced state, pressing upon the bronchi and root of the trachea.* "Such is the successful termination, (says Mr. W. in his report dated the 5th Dec.) of an operation, the principles of which seem to me to be now perfectly understood, and completely established." We shall abstain from all further comment on this very extraordinary case, until Mr. W. shall have either reconciled the assertion just quoted, with the present most deplorable state of the patient, or, by delaying to do so, shall have left the world at liberty to form its own conjectures. We shall look forward with much interest, to the publication of Dr. Barry's Notes, alluded to in his Letter (to the Lancet) of the 15th December.

N.B. In our Fasciculus, for January, 15th, we shall offer some observations on the operation for aneurism, ultra tumorem, and *fear* we shall be obliged to give the conclusion of Mrs. Denmark's case.

21*st Dec.* 1827.

---

* Lancet, No. 223.

† Lancet, No. 220.

